# Toward a pharmacy curriculum theory: spiral integration for pharmacy education

**DOI:** 10.5116/ijme.58a8.0381

**Published:** 2017-02-25

**Authors:** Nicole Rockich-Winston

**Affiliations:** 1 Department of Pharmacy Practice, Administration and Research, Marshall University School of Pharmacy, USA

**Keywords:** Pharmacy, curriculum theory, spiral integration, pharmacy education, USA

## Introduction

The explosion of cumulative new molecular entities approved by the United States Food and Drug Administration (FDA) has more than doubled in the last 30 years, from approximately 700 in 1985 to a staggering 1,539 as of December 31st, 2015.^1^^,^^2^ Pharmacists are expected to be the “drug experts” of the healthcare team, providing insight on ideal drug therapies, doses, formulations, side effects, drug interactions, and patient counseling for each approved drug. However, with the ever-increasing new drug classes and the fixed amount of time dedicated to the didactic pharmacy curriculum, scholars have offered a few solutions including flipped classroom and asynchronous learning to proactively address the magnitude of drug information as well as other important pharmacist-related knowledge that must be imparted to students during their didactic curriculum.^3^ On the other hand, these strategies may not necessarily address the limited “contact points” in a curriculum for lesser known drugs, which in turn decreases knowledge retention rates. So the central question is: how can one design a pharmacy curriculum to take into account the cumulative rate of FDA approved drugs and provide multiple contact points within the curriculum to increase retention of drug information? The answer lies within two curricular words: spiral integration.

### Historical and current perspectives on spiral integration

To begin, the concept of spiral curriculum was championed by Jerome Bruner in the mid-20th century. In his article, “The Process of Education” he describes how elementary students can learn to think like a physicist or mathematician, learning more “surface level” information in primary school, then progressing to more abstract concepts as students near graduation.^4^ Medical education has embraced Bruner’s spiral theory for many years.^5^^-^^7^ Harden and colleagues first described vertical and horizontal integration in medical education, which was later coined “spiral integration,” as learning basic and clinical sciences across time (vertical integration) and discipline (horizontal integration).^5^^,^^6^ More specifically, medical students learn both basic and clinical sciences throughout their didactic studies, which additionally incorporates clinical methods, ethics and health promotion.

In pharmacy education, Pearson and Hubball describe the efforts of the University of British Columbia to design and institute spiral integration into their curriculum.^8^  Horizontal integration is achieved through sequencing of courses and semester-long case-based tutorials while vertical integration is achieved through instructor collaboration.^8^ Similarly, Husband, Todd and Fulton discuss the curricular structure at Durham University and the University of Sutherland in greater detail, using the body systems approach to provide both horizontal and vertical integration.^9^ Although these programs begin to embrace the spiral integration model developed by Harden and Stamper, these pharmacy curricular designs described do not address the logical sequencing that medical curricular models have incorporated.^5^ The logical sequencing of spiral integration should provide increasing complexities in the curriculum. In each of the pharmacy curriculum models, however, students will be introduced to drug names, mechanisms of action, side effects, and counseling points late in their pharmacy studies. How, then, could this dilemma be addressed in a way that embraces the spiral integration model?

### Toward a pharmacy curriculum theory

We have come to accept that basic sciences and fundamental principles of drug discovery, action, and delivery provide the ideal foundation for pharmacy students. In medical education, the spiral integrated curriculum model begins with normal structure function and behavior of physiological systems, progressing to abnormal structure function, clinical practice, and ending with experiential training.^5^^-^^7^ As a result, medical students are introduced to the language and concepts of healthy and diseased systems, aiding in their ability to diagnose patients. As such, would it not make the most logical sense to introduce pharmacy students to the language and concepts of pharmaceutical drugs beginning on day one of the curriculum?  Some might argue that medicinal chemistry, biochemistry, and pharmaceutics provide this foundational language. However, if we look at the fundamental language for pharmacy students, it is drug names and associated characteristics, including doses, formulations, mechanism of action, and side effects. Once pharmacy students have mastered this information, the curriculum would progress to basic and clinical sciences, providing formal logical and applicable explanations, respectively.

As an example, in an ideal curriculum pharmacy students would initially learn the following information about rosuvastatin calcium:  rosuvastatin treats hyperlipidemia and hypertriglyceridemia, it is an inhibitor of the enzyme HMG-CoA reductase, it is available as an oral tablet in four strengths, which is typically dosed once daily, and it has common side effects of muscle pain, constipation and nausea.^10^ Later in the curriculum when students are introduced to cholesterol metabolism, the pathophysiology of hypercholesterolemia, and associated clinical applications, the student would be asked to recall the “basic” information of statins, including rosuvastatin. Some might still wonder how this is foundational knowledge for a pharmacy student. But ask yourself, how are drugs named? Pharmaceutical companies, academics and other researchers choose the scientific name of a new drug entity. Healthcare professionals associate the suffix–statin with cholesterol or possibly more specifically an inhibitor of HMG-CoA reductase, but does this suffix reveal anything about its chemical structure? Additionally, does the scientific name provide a logical explanation to the available dosage forms and associated strengths? Pharmacy educators and students might be better served if this drug knowledge could be horizontally integrated with the traditional foundational courses, with all drugs within a therapeutic class being adequately discussed, rather than using a few as examples. This alternative approach would still provide additional “contact” points in subsequent semesters when clinical aspects are discussed in greater detail. In both cases described, students would preferably master the majority of drugs available within the first year, learning the language of the pharmacy.

## Conclusions

As pharmacy curricula continue to evolve, the success of spiral integration in medical education offers insightful strategies to address the increasing number and complexity of FDA approved medications. Moreover, providing multiple contact points related to pertinent drug information supports the academy’s goal to develop pharmacists as drug experts. As such, there is significant promise in the spiral integration model with the language of pharmacy introduced early, increasing both the breadth and depth attainable during the didactic years. Clearly, the spiral integration model will need to be assessed to support its adoption by schools and colleges of pharmacy. Our hope for the pharmacy program at Marshall University in Huntington, West Virginia is that we will meet this evidentiary need, providing an evidence-based approach to support spiral integration in pharmacy education.

### Conflicts of Interest

The authors declare that they have no conflict of interest.
